# Is simultaneous bilateral total hip arthroplasty deleterious in a biomechanical point of view? A comparative gait analysis study

**DOI:** 10.1186/s12891-022-05856-y

**Published:** 2022-10-10

**Authors:** Martin Caudron, Christine Detrembleur, Maïté Van Cauter

**Affiliations:** 1 Université Catholique de Louvain, Cliniques Universitaires Saint-Luc, Service d’orthopédie et de traumatologie de l’appareil locomoteur, Avenue Hippocrate 10, B-1200 Brussels, Belgium; 2 Institut de Recherche Expérimentale Et Clinique, Neuro Musculo Skeletal Lab (NMSK), Université Catholique de Louvain, Secteur Des Sciences de La Santé, Avenue Mounier 53, B-1200 Brussels, Belgium; 3 Université Catholique de Louvain, Cliniques Universitaires Saint-Luc, Service d’orthopédie et de traumatologie de l’appareil locomoteur, Avenue Hippocrate 10, B-1200 Brussels, Belgium

**Keywords:** Hip osteoarthritis, Pain, Gait analysis, Biomechanics, Bilateral total hip arthroplasty

## Abstract

**Purpose:**

Uni- or bilateral hip osteoarthritis is a common disease generating pain, stiffness, and functional disabilities. Changes in the normal walking with higher energy expenditures are observed. Facing a cruel lack of biomechanical data, we decided to analyse the impact on the walking of single and simultaneous bilateral total hip arthroplasties (THA).

**Method:**

We conducted a prospective monocentric study, comparing two matched groups of 15 patients able to walk with symptomatic isolated uni- (group 1) or bilateral HO (group 2) and treated respectively by unilateral THA (UTHA) or simultaneous bilateral THA (SBTHA). Surgery was performed by a single surgeon with a direct anterior approach and approved by local ethical committee. Walking was assessed by a « 3D Gait analysis motion» pre and at 6 months post operatively.

**Result:**

In the UTHA group, recovery*, i.e., efficiency of locomotor mechanism (p* < *0.001)* and pelvis sagittal balance *(p* = *0.031)* improved, while external and total work *(p* = *0.010)* decreased post operatively. In the SBTHA group, speed (*p* = 0.035), step length (*p* = 0.046), range of motion of knee sagittal stance (*p* = *0.009*) and hip frontal (*p* = *0.031*), and internal work are significatively higher (*p* < *0.001*) post operatively.

**Conclusions:**

This original study attests that THA has a positive impact on walking and energetics outcome in UTHA and SBTHA.

## Introduction

Hip osteoarthritis (HO) is a chronic disease generating pain [1], disability, stiffness, and alteration in gait function [2]. It is established that osteoarthritis is the third most rapidly rising condition associated with disability [3]. According to Global health Metrics, HO affects 40 million people worldwide [4].

Diagnostic of a symptomatic HO may lead to total hip arthroplasty (THA) to improve gait function and quality of life. It has been proven that replacement of the coxo-femoral articulation alleviated pain and stiffness [5]. The goal of THA is to restore the normal pain free function of the hip. Studies are generally based on functional outcomes, range of motion (ROM) and radiological evaluation. It is well known that restoration of leg length, centre of rotation, acetabular and femoral offset increase implants survival and satisfaction [6].

THA is known as the surgery of the century for symptomatic HO [7]. Sequential THA for bilateral HO with generally a 3–6-month interval period is the most used procedure [8]. Since 1996, simultaneous bilateral total hip arthroplasty (SBTHA) is known to be a safe procedure with good outcomes and gave a real improvement in walking abilities in patient with bilateral and symptomatic HO [9].

Walking is a movement based on a cyclic activity: the walking cycle is characterized from a physical point of view by spatio-temporal, kinematic, kinetic, mechanical, and energetic elements [10, 11].

Thus far, most studies compared the effect of unilateral THA (UTHA) on biomechanics against healthy individuals**.** It has been described by Bahl & al., that patients with OA have a decrease of walking speed, step length, single-limb support time, sagittal and coronal plane hip range of motion (ROM) compared to health population [12]. The aim of this study is to evaluate changes in the walking in UTHA and SBTHA.

To date, with the development of minimal invasive surgery (MIS), it has been well established that SBTHA is safer than two-stage in patients with symptomatic bilateral HO [13, 14]. This procedure reduces the length of hospital stay and is cost effective [15]. Evidence base medicine requires meticulous assessment of treatment; in this sense the World Health Organization (WHO) has created an International Consortium for Health Outcomes Measurement (ICHOM). Patients are nowadays evaluated in their globality through validated questionnaires. Patient-reported outcome measure (PROMS) are valid and reproducible in hip registries such as the Short Form-36 Health Survey (SF-36) that is the most widely used health-related quality of life (QOL) measure in research to date [16]. Oxford Hip score (OHS) is another valid tool for self-assessment of pain and function [17]. Those easy-to-use scores are well spread in the literature and give indirect, though subjective, information about effectiveness of treatment. There are only few prospective comparative studies in the field of biomechanics and dynamic up to now. The lack of data in the literature limits the full integration of ICHOM because we need more objective, independent and dynamic measures to fully attest efficiency. With the technology available in our motion laboratory, it was mandatory to gain more information about SBTHA, to compare the procedure and its effects with the actual gold standard (UTHA). Motion lab system give mechanical, kinematic, kinetic, mechanic, and energetic values that will help us collecting experience data.

Our hypothesis is that SBTHA is offering a better balance motion recovery (because avoidance of protective contralateral reflexes) nevertheless with a higher energetic gait pattern request because of two site surgeries comparing to unilateral procedures.

## Patients and methods

### Study design

Between November 2015 and June 2020, we conducted a longitudinal prospective study in the orthopaedics department unit*.* The study was approved by the local ethics committee (*B403201523492*), and all patients gave written informed consent prior to participation. Patients in the first group underwent primary UTHA while the second group underwent SBTHA. The surgeries were conducted following a direct anterior approach (DAA) without traction table, by a same single operator, and using material from two different companies. Patients were assessed pre- and post-operatively (6 months ± 2 weeks) with questionnaire (OHS and SF-36) and with a three-dimensional gait lower limb assessment (3DGLA).

### Participants

Patients with single or bilateral symptomatic HO, older than fifty years old, able to walk for five minutes without assistive devices were recruited. Operative indication for primary THA was symptomatic and severe HO on plain X-rays of hip and pelvis. Dysplastic hips were excluded (Crowe III and IV). A first group of 20 patients with severe and isolated symptomatic unilateral HO was recruited between December 2015 and August 2016 (UTHA group). In a second time, five patients of the UTHA group were excluded after matching according to age, sex, and body mass index (BMI) with the second group. In UTHA group, 12 patients had Kellgren and Lawrence OA grade 3 or 4 only for isolated one hip without radiological sign of HO on the other side. Two patients had bilateral HO but only one symptomatic side. One patient had a previous THA on the other side few years ago. A second group of 15 patients with severe and symptomatic bilateral HO was then included between August 2017 and March 2020 (SBTHA group). Patients received information about the study from the surgeon himself, from an intern, and before 3DGLA. At baseline, both groups were homogenous as indicated in Table 1.

**Table 1:** Anthropometric data (mean (Standard deviation)) in unilateral HO vs bilateral HO groups at baseline

	Unilateral HO (*n* = 15)	Bilateral HO (*n* = 15)	*p*
Age (years)	69.4 (12.7)	62.9 (8.8)	0.117
Height (m)	1.72 (0.06)	1.75 (0.07)	0.304
Weight (kg)	84.23 (12.9)	82.63 (18.2)	0.784
BMI (%)	28.3 (4.3)	26.7 (4.8)	0.351
Sex (Male/Female)	12 / 3	12 / 3	1.000

### Functional assessments

Following ICHOM recommendations, pain and function were attested through OHS, and QOL with SF-36. These outcome measures are the most frequently used. It allows the investigator to have a rapid *snapshot* of the patient condition and its inherent inabilities in daily life [18].

### Three-Dimensional Gait lower limb assessment

**Fig. 1:**
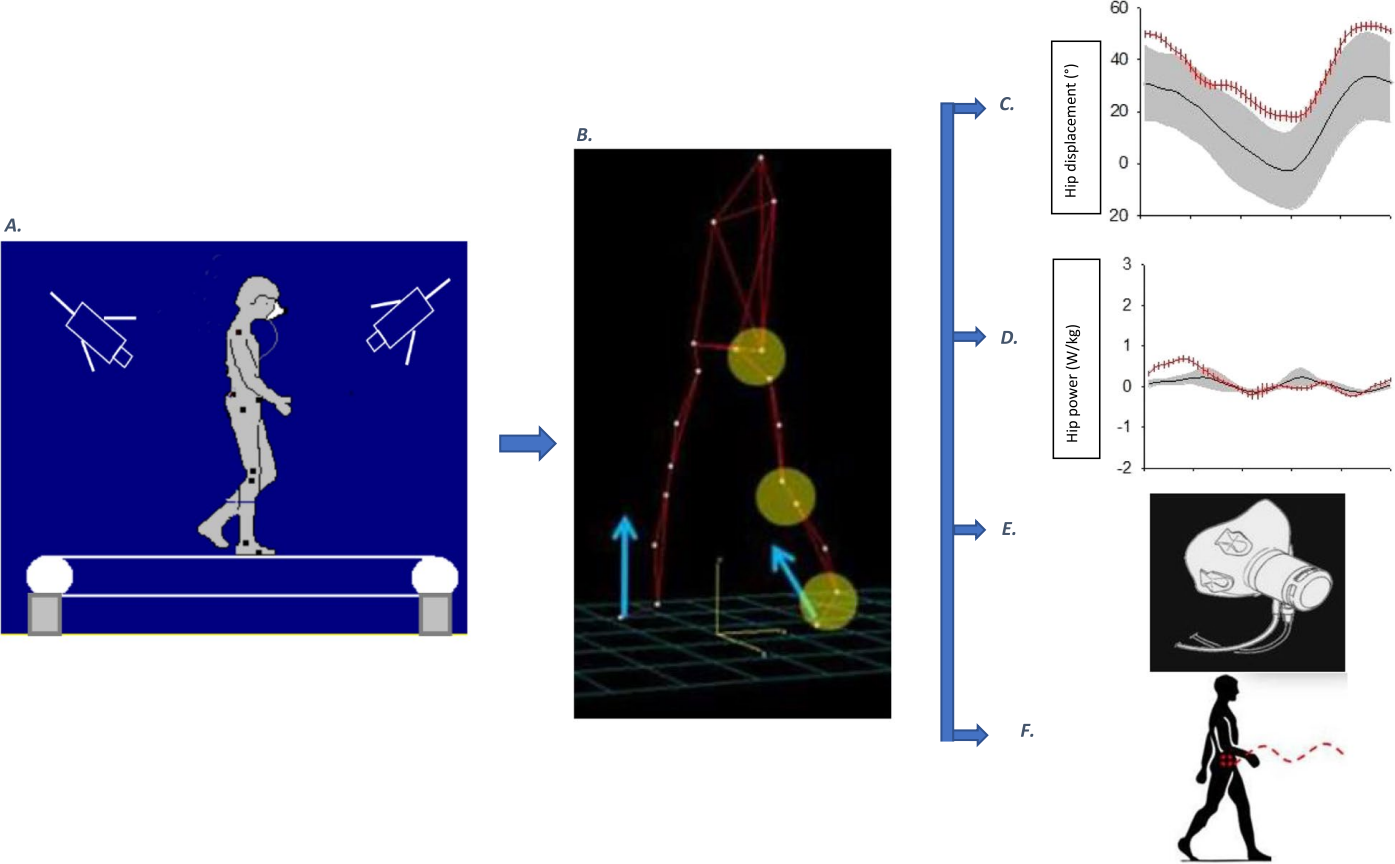
Gait analysis laboratory (**A**). Patient is equipped with a reflective marker captured by 8 infrared cameras, walks on a treadmill equipped with strain gauges and equipped with a bucco nasal mask. From the recorded signals (**B**), we calculate the angular displacement of the different segments (**C**), the muscular moment and power (**D**), the mechanical work and the energy cost (**E** & **F**). The curves shown in Fig. 1 C and D are those of the hip. The gray traces represent a normal trace (mean with standard deviation) and the red a patient in pre-treatment

**Fig. 2:**
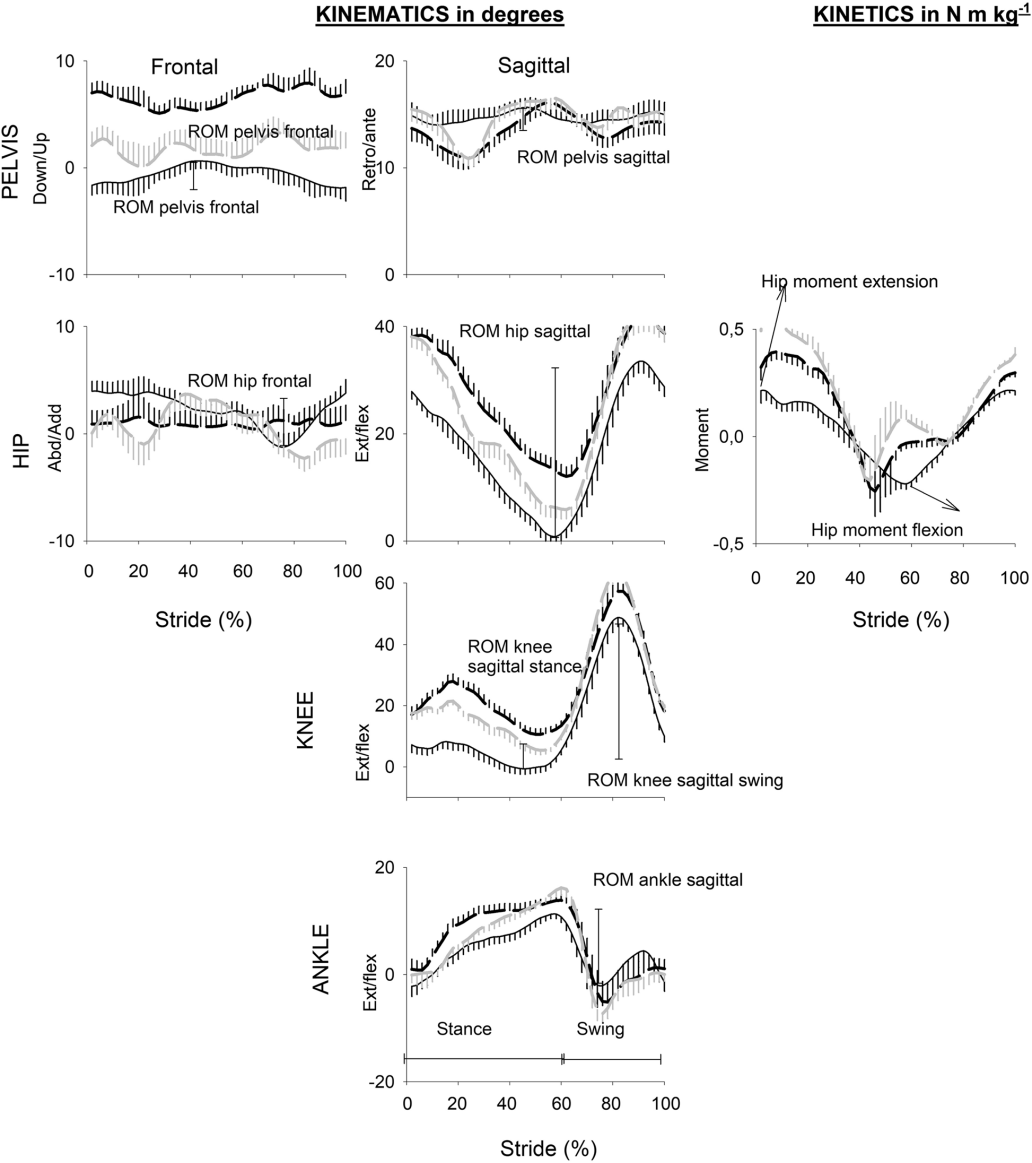
Evolution of kinematics (in degrees) and kinetics (in N m/kg) curves as function of normalised stride (in %) in healthy subject of 70 years walking at 3 km/h (continuous line); and in a SBTHA patient (dash black line in pre op and dash grey line in post op). Vertical bars represent standard deviation in one direction (posiyive or negative) 0% correspond to initial contact. The stance phase takes place from 0 to 60% and the swing phase from 60 to 100%. The range of motion (ROM) of kinematics calculated in this study are indicated on each graph

Patients were asked to walk on an instrumented treadmill at a self-selected comfortable speed during five minutes without any exterior help. Patients walked at their comfortable speed. The speed was chosen by the 10 m test on flat ground. The test is performed before each gait analysis. The speed of the treadmill is the one defined in the 10 m test. Participants were equipped with 19 reflective markers located on specific anatomical landmarks [19]. Eight infra-red cameras (Vintage V5, Vicon) located around treadmill, recorded 3D coordinates of markers at 100 Hz and 3D angular displacements calculated [19]. 3D strain gauges fixed under treadmill (100 Hz) recorded external forces. Participants wore a nasal mouth mask relied to ergospirometer recording oxygen consumption. From kinematics data, spatio-temporal parameters (speed, step length, cadence) were calculated. On each angular displacement curve in 3D plane (pelvis, hip, knee, and ankle), we measured the ROM, defined as peak-to-peak amplitude (Fig. 1). Kinematic data were normalized to 100% of the time of the stride, with 0% corresponding to the initial contact. From kinematics data and forces, hip moment of force in extension and flexion were calculated (Fig. 2).

The total muscular mechanical work (*Wtot*) was also assessed. It corresponds to the sum of the external work (*Wext*), i.e. the work performed by the muscles to move the center of body mass (COM) relative to the surroundings, and the internal work (*Wint*), i.e. the work performed by the muscles to move the body segments relatively to the COM [20]. The *Wext* was computed from 3D-ground reaction forces according to Cavagna [21]. The recovery quantifying the percent of mechanical energy saved by a pendulum-like exchange between the gravitational potential energy and kinetic energy of the COM. The *Wint* was computed from kinematics data measured with the motion capture system according to Willems [22]. All data were recorded during several strides, and data obtained during 10 consecutive strides were averaged. The mean values obtained were used for statistical analysis.

Each energy measurement started with a rest period in which the subject was standing on the treadmill. Thereafter, they walked until a steady state was reached and maintained for at least 2 min. The assessment of metabolic energy cost of gait was performed with an ergospirometer (Medisoft, Belgium) by measuring the subject’s oxygen consumption. The net energy cost was calculated as ‘the metabolic cost of walking minus the metabolic cost of standing’ divided by speed [23]. The efficiency was calculated as the ratio between *Wtot* and net energy cost.

### Statistical analysis

Data were analyzed using Sigmaplot V3 software package (SPSS Inc., Chicago, IL, USA). The variables were tested for normality using the Kolmogorov–Smirnov test. All data were presented as mean and standard deviation because they were normally distributed. First, demographic data were compared between groups (uni vs bilateral sides) using independent sample t test. Second, effect of surgery (pre- vs post-surgery) was tested in each group using t-paired test. Third, changes in outcomes for post vs pre-surgery assessments between both groups were compared using t-paired test. This test was used because we paired our patients to increase power of our results and avoid age or overweight bias. A *p* < 0.05 was considered as statistically significant.

## Results

In total, 30 patients, 46 arthroplastic hips and 60 hips were included. 26 participants passed all tests, 4 patients did not pass the post operatively 3DGLA, one because of time schedule issues, and 3 because of the *Covid-19* pandemic regulations. Mean was calculated to assess missing data of patients who did not pass post op 3DGLA because of pandemic regulations. Radiologically, all patients were classified Kellgren and Lawrence grade 3 or 4 on operated sides [24]. Patients were discharge from hospital when they can walk and climb stairs without exterior help. Any physiotherapy was given to patients outside hospital, so they were all able to perform a self-rehabilitation.

Data in the UTHA group are presented in Table 2, data in the SBTHA in Table 3 and changes comparing both groups (UTHA vs SBTHA) in Table 4.

**Table 2:** Effect of treatment pre vs post operatively in the unilateral group

		*PRE* *MEAN (SD)*	*POST* *MEAN (SD)*	*p*
***Spatio temporal***	Speed (km/h)	2.58 (0.81)	2.54 (0.85)	0.844
	Step length (m)	0.451 (0.1)	0.468 (0.07)	0.587
	Cadence (step/min)	106.5 (13.2)	102.5 (16.9)	0.236
***Kinematics***	ROM pelvis sagittal (°)	4.06 (0.9)	3.34 (1.1)	0.031*
	ROM hip sagittal (°)	28.54 (8.7)	33.31 (6.4)	0.052
	ROM knee sagittal stance (°)	2.16 (4.7)	1.08 (4.6)	0.379
	ROM knee sagittal swing (°)	39.27 (13.3)	44.13 (9.6)	0.111
	ROM ankle sagittal (°)	18.37 (5.5)	18.15 (4.1)	0.894
	ROM pelvis frontal (°)	4.84 (2.6)	4.16 (1.3)	0.292
	ROM hip frontal (°)	7.52 (4.4)	7.62 (2.8)	0.923
	ROM pelvis transverse (°)	5.36 (2.9)	6.08 (3.1)	0.284
***Kinetics***	Hip Moment extension (N m/kg)	0.424 (0.13)	0.46 (0.18)	0.403
	Hip Moment flexion (N m/kg)	- 0.281 (0.18)	- 0.37 (0.25)	0.183
***Mechanics***	External work (J/kg m)	0.581 (0.31)	0.357 (0.21)	0.010*
	Internal work (J/kg m)	0.195 (0.04)	0.198 (0.05)	0.824
	Total work (J/kg m)	0.776 (0.29)	0.555 (0.21)	0.007*
	Recovery (%)	26.2 (15.6)	46.6 (17.4)	< 0.001*
***Energetics***	Cost (J/kg m)	2.934(0.83)	2.981 (0.52)	0.791
	Efficiency (%)	24.18 (7.6)	20.64 (11)	0.346
*Oxford score (/48)*		24.8 (9.2)	41.6 (5.1)	< 0.001*
*SF36-PC (%)*		34.8 (7.3)	49.6 (6.8)	< 0.001*
*SF36-MC (%)*		44.6 (9.8)	55.5 (4.2)	0.006*

*Legend*: values are expressed as mean and standard deviation (SD). Speed values are in kilometres per hour (km/h), step length in meter (m), cadence in step per minute (step/min). Kinematics values are in degrees (°). Kinetics values in Newton meter per kilogram (Nm/Kg). Mechanics values in joules per kilogram meter (J/Kg.m). Recovery and efficiency in percentage (%). Note: ( ∗) indicates significant differences between PRE-MEAN and POST-MEAN (*p* < 0.05)

**Table3:** Effect of treatment pre vs post operatively in the bilateral group; values are expressed in mean with SD

		*PRE* *MEAN (SD)*	*POST* *MEAN (SD)*	*2 SIDES* *p pre vs post*
***Spatio temporal***	Speed (km/h)	2.7 (1)	3.3 (0.55)	0.035*
	Step length (m)	0.495 (0.15)	0.575 (0.07)	0.046*
	Cadence (step/min)	101.3 (14.4)	106.5 (8.6)	0.318
***Kinematics***	ROM pelvis sagittal (°)	3.94 (1.3)	3.92 (1.1)	0.921
	ROM hip sagittal (°)	33.56 (8.2)	37.67 (4)	0.072
	ROM knee sagittal stance (°)	2.74 (5.7)	6.61 (5.5)	0.009*
	ROM knee sagittal swing (°)	46.51 (9.1)	50.29 (6.9)	0.091
	ROM ankle sagittal (°)	20.91 (6.7)	23.12 (5.2)	0.166
	ROM pelvis frontal (°)	5.01 (2.5)	5.69 (2.3)	0.424
	ROM hip frontal (°)	7.68 (2.8)	9.58 (3.6)	0.031*
	ROM pelvis transverse (°)	6.23 (2.4)	6.31 (1.5)	0.921
***Kinetics***	Hip Moment extension (N m/kg)	0.6 (0.26)	0.582 (0.21)	0.833
	Hip Moment flexion (N m/kg)	- 0.561 (0.32)	- 0.388 (0.27)	0.167
***Mechanics***	External work (J/kg m)	0.389 (0.31)	0.272 (0.05)	0.199
	Internal work (J/kg m)	0.196 (0.04)	0.265 (0.06)	< 0.001*
	Total work (J/kg m)	0.588 (0.29)	0.535 (0.09)	0.564
	Recovery (%)	49.1 (21.1)	55.7 (11.8)	0.248
***Energetics***	Cost (J/kg m)	3.253 (0.85)	2.974 (0.62)	0.364
	Efficiency (%)	18.16 (3.8)	18.7 (5)	0.694
*Oxford score (/48)*		18.2 (6.6)	45.9 (1.8)	< 0.001*
*SF36-PC (%)*		43.3 (16.9)	77.3 (11.4)	< 0.001*
*SF36-MC (%)*		45.2 (18.1)	75.3 (10)	< 0.001*

**Table 4:** Effect of surgery in the unilateral group compared to the bilateral group; values are expressed in mean with standard deviation (SD)

		*Unilateral* *MEAN (SD)*	*Bilateral* *MEAN (SD)*	*p*
***Spatio temporal***	Speed (km/h)	-0.03 (0.65)	0.593(0.96)	0.031*
	Step length (m)	-0.01 (0.159)	0.08 (0.14)	0.131
	Cadence (step/min)	-10.9 (31.3)	4.7 (17.1)	0.116
***Kinematics***	ROM pelvis sagittal (°)	-0.73 (1.1)	-0.28 (1.5)	0.377
	ROM hip sagittal (°)	6.7 (12.1)	4.1 (8.3)	0.398
	ROM knee sagittal stance (°)	-0.9 (4.4)	2.7 (6.3)	0.097
	ROM knee sagittal swing (°)	7.5 (14.5)	0.3 (15.3)	0.124
	ROM ankle sagittal (°)	-0.2 (6.7)	-0.2 (10.9)	0.994
	ROM pelvis frontal (°)	-0.7 (2.3)	0.3 (3.5)	0.459
	ROM hip frontal (°)	0.1 (3.9)	1.9 (3.1)	0.248
	ROM pelvis transverse (°)	1.1 (2.5)	-0.3 (3.5)	0.083
***Kinetics***	Hip Moment extension (N m/kg)	0.03 (0.16)	-0.05 (0.46)	0.491
	Hip Moment flexion (N m/kg)	- 0.09 (0.24)	0.14 (0.48)	0.132
***Mechanics***	External work (J/kg m)	-0.23 (0.29)	-0.12 (0.33)	0.374
	Internal work (J/kg m)	0.003 (0.05)	0.07 (0.06)	0.011*
	Total work (J/kg m)	-0.22 (0.27)	-0.05 (0.34)	0.160
	Recovery (%)	20.3 (16.2)	6.5 (20.3)	0.095
***Energetics***	Cost (J/kg m)	-0.35 (1.17)	0.15 (2.16)	0.345
	Efficiency (%)	-3.1 (15.8)	4.1 (9.4)	0.170
*Oxford score (/48)*		16.7 (9.9)	27.3 (19.3)	0.119
*SF36-PC (%)*		14.8 (10.1)	35.6 (32.1)	0.012*
*SF36-MC (%)*		10.9 (10.2)	28.7 (35.2)	0.078

In the unilateral group, pelvis sagittal balance *(p* = *0.03)* is significatively improved, while a significative decrease of *Wtot* and *Wext (p* = *0.01)* is observed with a better recovery *(p* < *0.001)*. Functional outcomes scores are significatively improved *(p* < *0.001). Note also that the only patient with previous THA on one side, was beyond the UTHA mean pre and post operatively and therefore does not influence the results.*

In the bilateral group (SBTHA), speed (*p* = 0.03) and step length (*p* = 0.04) are improved. ROM of Knee sagittal stance (*p* = 0.009), and ROM hip frontal (*p* = 0.03) increase. The *Wint* is significatively higher (*p* < 0.001). Functional outcome scores are significatively better (*p* < 0.001).

The changes expressed as “*post minus pre values*” are significantly improved in favour of bilateral group for speed and SF36 PC. The change seems in disfavour of bilateral group for *Wint* but attributed to an increase of speed in this group.

## Discussion

The study is to our knowledge the first one comparing UTHA and SBTHA based on biomechanical and energetics fields. Data were collected preoperative and at 6 months postoperative by a same high volume hip surgeon, with a single DAA without traction table. This study gains in interest as DAA became more frequently used nowadays, furthermore up to now we did not have any biomechanical information in a DAA SBTHA cohort at 6 months post operatively. Our results attest that surgery has a positive impact in both PROM’s score, biomechanical and energetic fields in both groups. Our hypothesis that SBTHA would generate higher energetic cost is rejected. SBTHA is therefore not deleterious to the patient from a biomechanical and energetical point of view.

Concerning the UTHA group, patients walked pre- and post-operatively at a relatively similar speed, as demonstrated in Colgan et al. [25], however the *Wtot* and *Wext* decreased as recovery improved significatively. This may be explained by a reduction in pelvic sagittal ROM. We conclude in this population, that surgery improves the pain free ROM of the hip, therefore patients do not need any extra production of *Wext* to produce avoidance limping as detailed by van Drongelen et al. [26]. In fact, as the hip becomes pain free, a better flexion of the hip and the knee is balanced through a better pelvic sagittal ROM. Energetic equilibrium between potential and kinematic energy is improved. Restoration of the oscillation of the COM will decrease the *Wext* needed to produce limping because of pain, stiffness, or both. The whole will allow a better gait pattern and spare mechanical energetics outcome at a same speed. Patients at last, can walk at a same self-selected comfortable speed with a normalized mechanical gait pattern post operatively. This correlates the work of Queen et al. attesting that avoidance of compensatory mechanisms increases hip power on the surgical sides and decreased in the non-surgical side, the whole automatically goes with a decreased in *Wext* [27].

About kinematics and kinetics parameters, our results are partially in agreement with Rathod et al. which observed at 6 months postoperatively, improvement in flexion/extension ROM, peak of flexion and extension moments. Our values are also improving but not significantly explained by the size of our sample [28].

Concerning the SBTHA group, patient spontaneously walks at a higher speed with a longer step length, mobility of both hips and knees improves gradually. These results are in agreement with Temporiti et al. [29]. Energetics exchange in *Wext* and *Wint* is foreseen at an equivalent *Wtot*. Alleviating pain and stiffness of both hips is having a huge impact on the knee sagittal stance. It is here one of the key elements to be encountered.

Before surgery, patients walk with stretched legs, because of pain and stiffness of hips, short step length, and a high transverse ROM pelvis. *Wext* is required to compensate lost kinetics energy from one leg to the other as the COM oscillates more laterally than vertically diminishing exchange in potential energy.

After SBTHA, for a same transverse ROM pelvis, step length and speed are increased. In the meantime, the knee stance phase is considerably increased, which attests that patient flex legs much more than preoperatively, just as the hip ROM increases too. Therefore, flexing hips and knees will decrease the necessity of energy output to upper the COM to help passing energy from one leg to the other.

At last, we may say, that patients walk faster, with better mobility closer to normal values, and better energetics distribution. The whole goes in parallel with good PROM’s which attests patients’ satisfactory.

In our population, ROM pelvis sagittal remains equal in our second group. Those measures differ from the work of Milan university attesting a pelvic kinematic profile closer to normative data was found in bilateral patients [30]. This might be explained by two distinct parameters: first, patients are evaluated at six months in our study versus seven days, which might allow patients to adapt to the hip replacement, and second, surgery approaches were different between both studies (DAA vs postero-lateral).

### Comparing groups

Unexpectedly, the groups differ from speed, *Wint* and SF36-PC. Which is mainly explained by the fact that SBTHA group walks faster, which increases the *Wint*. Therefore, we may conclude that the total energetic cost is similar in both groups. The *gait pattern* may still differ from a healthy population but has been improved post operatively as shown by Bahl & al [12]. Several study protocols are based at a same and fixed speed for all participants, we decided here to let patients walks at their own speed preferences, to avoid any distributions changes dues to an unusual speed specific patient. UTHA meta-analysis suggests improvement but subsidence of functional limitation according to healthy population [31], data here suggests that SBTHA patients have a similar benefit than UTHA. In parallel, PROM such as OHS and SF-36 confirms that patients are improved in most aspects of their own QOL, and our data are similar to national registry [32].

*Limitations* in our study need to be considered. First, this study compares two different populations without randomization and small group size. Some patients selected in the SBTHA group were not included for several reasons such as time schedule issues, no interest, no available place in our gait lab. Four patients did not pass the postoperative 3DGLA because of *Covid-19* pandemic regulations. Patients in this group are younger and more active. Several patients also preferred a two-stage surgery. No severe adverse events were observed in our cohort. We decided not to include patients in two stage THA surgery to avoid interpretations bias in 3DGLA.

In conclusion, SBTHA is known to be the safest and cost saving procedure in a population with symptomatic bilateral HO that improves QOL as proven with PROM’s according to ICHOM standards [33, 34]. We may now attest, based on a walking laboratory analysis at 6 months post operatively, in a DAA without traction table cohort study, that SBTHA produces a similar gait pattern, an optimal recovery, and a non-excessive energetic cost compared to UTHA in unilateral HO. Despite thoughts, energetics cost in walking in SBTHA is not modified and may be due to avoidance of compensatory mechanisms through normalisation of the walking and thus has no adverse impact on rehabilitation compared to UTHA.

## Data Availability

The datasets used and/or analysed during the current study are available from the corresponding author on reasonable request.
